# Frequency of Radiologic Bronchiolocentric Interstitial Pneumonia Patterns and Associated Factors in Welders

**DOI:** 10.1155/carj/3335338

**Published:** 2026-09-28

**Authors:** Yusuf Samir Hasanlı, İpek Candemir, Yeliz Aktürk

**Affiliations:** ^1^ Occupational Diseases Unit, Etlik City Hospital, Ankara, Türkiye; ^2^ Department of Pulmonology, Etlik City Hospital, Ankara, Türkiye; ^3^ Department of Radiology, Etlik City Hospital, Ankara, Türkiye

**Keywords:** bronchiolocentric interstitial pneumonia, hypersensitivity pneumonitis, occupational exposure, radiologic pattern, welding fumes

## Abstract

**Background:**

Bronchiolocentric interstitial pneumonia (BIP) is an airway‐centered pattern observed in various interstitial lung diseases. This study aimed to evaluate the clinical, occupational, and radiological factors associated with the frequency of the BIP pattern on HRCT in welders.

**Methods:**

In this retrospective cross‐sectional study, an operational HRCT‐based methodology was used to systematically define the BIP pattern in 73 male welders. The frequency of the BIP pattern and its associations with clinical, demographic, and occupational factors were investigated by comparing cases according to BIP status. Cases were compared according to the presence of BIP, which was defined based on operational radiological criteria. Appropriate parametric and nonparametric tests were used for statistical analyses.

**Results:**

The BIP pattern was detected in 21.9% of welders (*n* = 16; 13 nonfibrotic and 3 fibrotic). Although BIP frequency did not differ significantly between never‐smokers and current smokers (13.3% vs. 16.2%; *p* = 0.102), cumulative smoking exposure was higher among BIP‐positive cases (32.5 vs. 20 pack‐years; *p* = 0.021). DLCO and KCO were significantly lower in BIP‐positive cases among the 33 patients with available diffusion capacity measurements (*p* = 0.007 and *p* = 0.002, respectively). Cases with the BIP pattern also had higher mMRC dyspnea scores (*p* = 0.032). The most frequent HRCT findings were bronchiolar wall thickening and centrilobular ground‐glass nodules.

**Conclusion:**

The BIP pattern is observed at a notable frequency on HRCT in welders. The findings suggest that BIP is not specific to hypersensitivity pneumonitis and may represent a morphological pattern associated with various inhalational etiologies. Occupational history should be carefully assessed in cases exhibiting the BIP pattern.

## 1. Introduction

Accurate identification of radiologic and pathologic patterns is essential for diagnosing interstitial lung diseases (ILD) and guiding management. In recent years, international guidelines have increasingly emphasized the recognition of patterns centered on small airways and bronchiolocentric distribution, beyond established interstitial pneumonia patterns such as usual interstitial pneumonia (UIP) and nonspecific interstitial pneumonia (NSIP) [1, 2]. This approach provides a novel perspective, particularly in the assessment of exposure‐related lung diseases. In this context, bronchiolocentric interstitial pneumonia (BIP) is defined not as a specific disease entity but as a radiologic and pathologic pattern reflecting airway‐centered interstitial involvement [1]. Although the BIP pattern has been most frequently associated with hypersensitivity pneumonitis (HP) in the current literature, it has been reported that this pattern is not specific to a single etiology and may also arise secondary to connective tissue disease–related conditions, drug exposures, or other inhalational insults [1, 3]. However, the etiological diversity and clinical correlates of the BIP pattern have not yet been fully elucidated.

Occupational exposure to welding fumes is an important risk factor, as welding fumes contain various metals and metal oxides, inorganic particles, gases, and ultrafine aerosols that can accumulate in small airways such as terminal and respiratory bronchioles, as well as alveolar ducts and sacs, leading to bronchiolocentric injury [4, 5]. Welders are therefore at high risk for a variety of occupational lung diseases, including pulmonary siderosis, asthma, chronic obstructive pulmonary disease, bronchiolitis, and small airway disease [4, 6]. However, studies systematically addressing radiologic findings of chronic welding fume exposure—such as centrilobular ground‐glass nodules, mosaic attenuation, and peribronchiolar ground‐glass opacities—within the BIP pattern are very limited.

This study aimed to determine the frequency of the BIP pattern on high‐resolution computed tomography (HRCT) in welders. Additionally, the associations of the BIP pattern with age, smoking history, duration of exposure, pulmonary function tests (PFTs), clinical findings, and occupational health and safety measures were evaluated. Through this approach, the study seeks to contribute to the understanding of the clinical and radiologic significance of the BIP pattern in the context of occupational exposures.

## 2. Materials and Methods

### 2.1. Study Design

This study was a retrospective, cross‐sectional observational study employing an operational HRCT‐based methodology to systematically define the BIP pattern. HRCT findings of welders were evaluated to determine the frequency of BIP and to investigate associated clinical, demographic, and occupational factors. BIP‐positive and BIP‐negative cases were compared regarding demographic characteristics, smoking history, duration of occupational exposure, working conditions, and PFT results. The study was reported in accordance with the Strengthening the Reporting of Observational Studies in Epidemiology (STROBE) guidelines.

### 2.2. Study Population

The study population was drawn from a total of 405 patients who presented to the Occupational Diseases Outpatient Clinic of Etlik City Hospital between December 2024 and December 2025. Among these, 83 were welders. Thus, this population represents patients presenting to an occupational diseases outpatient clinic rather than a representative sample of the general labor force. Due to the retrospective design, all welder patients who presented during the specified period and met the eligibility criteria were considered for the study. After applying the exclusion criteria, 73 welder patients were ultimately included (Figure 1).

**FIGURE 1:**
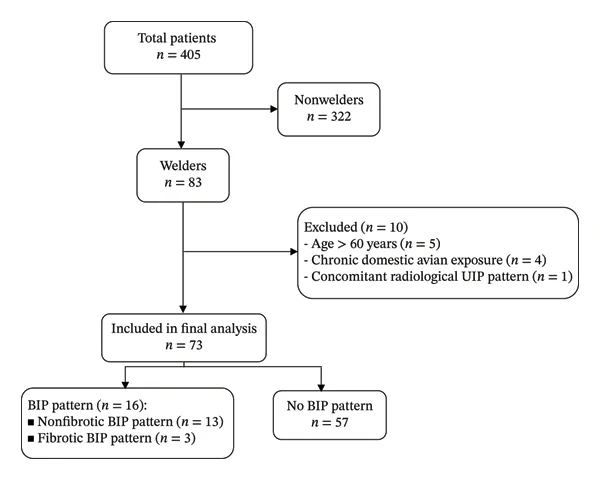
Flowchart of study population selection. BIP: bronchiolocentric interstitial pneumonia; UIP: usual interstitial pneumonia.

### 2.3. Inclusion and Exclusion Criteria

Inclusion criteria were male sex, age 18–60 years, at least one year of active welding experience, availability of HRCT, and complete clinical and sociodemographic data. The upper age limit of 60 years was selected to reduce heterogeneity related to cessation of occupational exposure around retirement age, as prolonged cessation of welding fume exposure may allow partial regression of some exposure‐related radiological findings.

Exclusion criteria were defined as being outside the specified age range; female sex due to insufficient numbers; presence of active pulmonary infection or aspiration pneumonia; malignancy; diagnosis of connective tissue disease or sarcoidosis; use of medications associated with pulmonary toxicity; presence of other ILD patterns (e.g., UIP or NSIP), whether previously diagnosed or not; and a prior diagnosis of HP or a history of other environmental or occupational antigen exposures strongly suggestive of HP. It should be noted that welding fumes themselves, including potential exposure to coatings or paints on metal surfaces, may also play a role in the etiology of HP. Patients with clinically or radiologically established nodular pneumoconiosis, including silicosis or silicosiderosis, were excluded. In particular, patients with a detailed occupational history indicating substantial silica exposure and characteristic HRCT findings, such as well‐defined centrilobular micronodules and/or progressive massive fibrosis, were excluded.

### 2.4. Data Collection

Data were retrospectively obtained from the hospital electronic records system. For all cases, sociodemographic characteristics, presenting complaints and physical examination findings, smoking status, duration of occupational exposure, working conditions, type of welding and welded materials, potential coexposures including grinding, painting, metal processing, and contact with metalworking fluids, use of personal protective equipment (PPE), PFT, and clinical diagnoses were recorded. Detailed occupational histories were reviewed to identify potential alternative inhalational exposures, particularly those potentially associated with HP.

### 2.5. Examined Parameters

The variables examined in the study included age, body mass index (BMI), smoking status (never, former, current, and pack‐years), symptoms (dyspnea [Modified Medical Research Council, mMRC] and cough), duration of welding work (years), type of welding used (gas metal arc welding [GMAW], electrode, and others [oxy‐acetylene, argon]), welded material (rusty iron, galvanized steel, and stainless steel), presence of an exhaust ventilation system, use of PPE, PFT parameters (forced vital capacity [FVC], forced expiratory volume in one second [FEV_1_], carbon monoxide diffusion capacity [DLCO], and carbon monoxide transfer coefficient [KCO]), and HRCT findings.

### 2.6. CT Examination and Radiological Assessment

Thoracic CT examinations were evaluated by consensus between an experienced thoracic radiologist and a pulmonologist using images obtained with a high‐resolution protocol. In the radiological assessment, findings with bronchiolocentric distribution were recorded separately as present or absent. The evaluated radiological findings included centrilobular ground‐glass nodules, bronchiolar wall thickening (assessed visually without quantitative measurement), mosaic attenuation, lobular air trapping, peribronchovascular ground‐glass areas, traction bronchiectasis, and the three‐density sign.

Due to the absence of a widely accepted CT diagnostic combination for the BIP pattern in the literature, an operational classification was used in this study to enhance diagnostic specificity, based on the typical HP HRCT pattern defined in the 2020 ATS/ALAT/JRS guidelines [7]. Accordingly, the BIP pattern was considered positive based on a specific combination of radiological findings with bronchiolocentric distribution (Table 1). To enhance diagnostic specificity, considering the nonspecific nature of isolated findings, the presence of at least one supportive finding alongside a mandatory finding was required; findings observed in isolation were not considered indicative of the BIP pattern. Cases with the BIP pattern were further divided into two subgroups based on the presence of accompanying fibrotic changes. Nonfibrotic BIP was defined by the presence of centrilobular ground‐glass nodules without fibrotic changes, with additional supportive findings potentially including mosaic attenuation and/or lobular air trapping. Fibrotic BIP was defined by the presence of traction bronchiectasis, with additional supportive findings potentially including peribronchovascular ground‐glass opacities, mosaic attenuation, lobular air trapping, or the three‐density sign (one or more). The potential radiological overlap between fibrotic BIP and other fibrotic interstitial pneumonia patterns was also considered. Among the three patients classified as having fibrotic BIP in our cohort, one had traction bronchiectasis, the three‐density sign, and mosaic attenuation, whereas the other two had traction bronchiectasis, peribronchovascular ground‐glass opacities, and mosaic attenuation. Therefore, the classification of fibrotic BIP was not based on the presence of a single radiological finding. The rationale for selecting centrilobular ground‐glass nodules as the mandatory finding in nonfibrotic BIP and traction bronchiectasis in fibrotic BIP was that these findings were considered the most appropriate and reproducible markers for operational pattern identification; centrilobular ground‐glass nodules reliably indicate inflammatory bronchiolocentric involvement in nonfibrotic BIP, whereas traction bronchiectasis reflects fibrotic distortion of the airways, making it the most specific marker for pattern determination in fibrotic BIP. Nonfibrotic and fibrotic BIP findings were recorded and analyzed separately.

**TABLE 1:** Operational BIP pattern and the reference criteria for typical HP on HRCT.

*A. Operational BIP pattern used in this study*		
BIP pattern	Mandatory findings	Associated/supportive findings^∗^
Nonfibrotic BIP	Centrilobular ground‐glass nodules	Mosaic attenuation and/or lobular air trapping
Fibrotic BIP	Traction bronchiectasis	Peribronchovascular ground‐glass opacities, and/or mosaic attenuation, and/or lobular air trapping, and/or the three‐density sign

*B. Reference typical HP HRCT pattern (ATS/ALAT/JRS 2020)*		
Typical HP subtype	Definition	
Nonfibrotic HP	Defined by the concomitant presence on HRCT of at least one small airway involvement feature (mosaic attenuation, air trapping, or the head‐cheese pattern) and at least one interstitial/parenchymal involvement feature (ground‐glass opacities or centrilobular nodules)	
Fibrotic HP	Defined by the presence on HRCT of fibrotic changes (irregular linear opacities, coarse reticulation, lung architectural distortion, and traction bronchiectasis or honeycombing) together with at least one small airway feature (ill‐defined centrilobular nodules, mosaic attenuation, the three‐density pattern, or air trapping)	

*Note:* Since there is no standardized CT diagnostic combination for BIP, the ATS/ALAT/JRS 2020 typical HP HRCT pattern, which defines the radiological correlate of bronchiolocentric involvement, was used as the reference when establishing the operational classification.

Abbreviations: BIP, bronchiolocentric interstitial pneumonia; HP, hypersensitivity pneumonitis.

^∗^At least one supportive finding is required.

### 2.7. Ethics Committee Approval

The study was approved by the Ethics Committee of Ankara Etlik City Hospital (approval no: AEŞH‐BADEK1‐2026‐071, Date: January 21, 2026). Due to its retrospective design, individual written informed consent was not required. The study was conducted in accordance with the Declaration of Helsinki and institutional ethical standards.

### 2.8. Statistical Analysis

Statistical analyses were performed using IBM SPSS Statistics Version 27.0. Continuous variables were expressed as mean ± standard deviation and 95% confidence interval (CI) for normally distributed data, and as median (interquartile range, IQR) for nonnormally distributed data. Categorical variables were presented as numbers and percentages (%). The mMRC dyspnea score was dichotomized as 0 and ≥ 1, and duration of welding work was categorized as ≤ 20 years and ≥ 21 years for categorical comparisons. Normality of the data was assessed using the Kolmogorov–Smirnov test, and homogeneity of variances was evaluated with Levene’s test. For comparisons between groups, an independent‐samples *t*‐test was used for normally distributed numerical variables, and the Mann–Whitney *U* test for nonnormally distributed numerical variables. The chi‐square test was applied for categorical variables. A *p* value of < 0.05 was considered statistically significant.

## 3. Results

As the main finding of the study, the BIP pattern was detected in 21.9% of patients (*n* = 16), with 13 cases exhibiting nonfibrotic and 3 cases exhibiting fibrotic features. The mean age of all 73 male patients was 47.5 years. The median cumulative smoking exposure was 20 pack‐years, and the mean duration of welding work was 25 years. PFT showed generally preserved spirometric parameters, while diffusion capacity parameters were reduced in the study population. Overall, 50.7% were current smokers; 54.8% had an mMRC score of 0; 43.8% reported cough; 95.9% worked indoors; and 64.4% welded without exhaust ventilation. GMAW was the most frequently used type of welding, and rusty iron was the most commonly welded material. The majority of workers either did not use protective masks at all or used them irregularly during welding. Concomitant asthma or COPD was present in 31.5% of cases. HRCT evaluation revealed at least one abnormal radiological finding in 68.5% of patients. The most commonly observed findings were bronchiolar wall thickening (47.9%) and centrilobular ground‐glass nodules (42.5%). Additionally, bronchoscopy and bronchoalveolar lavage were performed for specific diagnostic evaluation in 7 of the 16 cases with a BIP pattern. Bronchoalveolar lavage samples demonstrated abundant siderophages with strong Prussian blue positivity, findings consistent with pulmonary siderosis associated with small airway disease (bronchiolitis). Detailed data are presented in Table 2.

**TABLE 2:** Distribution of continuous and categorical variables in the study population.

Variables	Category/summary	*n*	%/Mean ± SD/median (IQR)	Min–max	%95 CI
Continuous variables					
Age (years)	Mean ± SD	73	47.5 ± 9.2	19–60	45.3–49.6
BMI (kg/m^2^)	Mean ± SD	70	27.8 ± 3.9	18.0–41.5	26.9–28.7
Smoking exposure (pack‐years)	Median (IQR)	58	20 (21)	1–70	
Duration of welding exposure (years)	Mean ± SD	73	25.1 ± 10.7	4–45	22.7–27.7
FVC (%)	Median (IQR)	69	96 (21)	19–134	
FEV1 (%)	Median (IQR)	69	89 (27)	16–129	
FEV1/FVC (%)	Median (IQR)	69	77.6 (9.8)	40.1–93.1	
FEF25‐75 (%)	Mean ± SD	69	72 ± 29	6–163	65–79
DLCO (%)	Mean ± SD	33	71 ± 22	20–137	63–79
KCO (%)	Mean ± SD	33	85 ± 19	23–131	78–92
Categorical variables					
Smoking status	Never smoker Former smoker Current smoker	15 21 37	20.5 28.8 50.7		
mMRC dyspnea score	0 1 2 Other	40 23 10 0	54.8 31.5 13.7 0.0		
Cough	No Yes	41 32	56.2 43.8		
Type of welding	Gas metal arc welding (GMAW) Electrode welding Other (oxyacetylene, argon welding)	47 19 7	64.4 26.0 9.6		
Material being welded	Rusted iron/steel Galvanized/stainless steel Both	51 4 18	69.9 5.5 24.6		
Indoor work environment	Yes No	70 3	95.9 4.1		
Local exhaust ventilation (LEV)	Present Absent	26 47	35.6 64.4		
Respiratory protective mask	Never used Irregular use Regular use	49 17 7	67.1 23.3 9.6		
Concurrent asthma/COPD	Yes No	23 50	31.5 68.5		
At least one abnormal CT finding	No Yes	23 50	31.5 68.5		
Centrilobular ground‐glass nodules	Yes No	31 42	42.5 57.5		
Mosaic attenuation	Yes No	6 67	8.2 91.8		
Areas of lobular air trapping	Yes No	23 50	31.5 68.5		
Peribronchovascular ground‐glass areas	Yes No	12 61	16.4 83.6		
Traction bronchiectasis	Yes No	3 70	4.1 95.9		
Tree‐density sign	Yes No	1 72	1.4 98.6		
Bronchiolar wall thickening	Yes No	35 38	47.9 52.1		
Nonfibrotic BIP pattern	Yes No	13 60	17.8 82.2		
Fibrotic BIP pattern	Yes No	3 70	4.1 95.9		
BIP pattern (nonfibrotic + fibrotic)	Yes No	16 57	21.9 78.1		
Specific diagnosis in cases with a detected BIP pattern	No further investigations performed for diagnosis	9	56		
	Pulmonary siderosis	7	44		

*Note:* Continuous variables with a normal distribution are presented as mean ± standard deviation (SD); for these variables, 95% confidence intervals (CIs) were additionally calculated. Continuous variables without a normal distribution are expressed as median (interquartile range, IQR).

Abbreviations: BMI, body mass index; DLCO, diffusing capacity of the lung for carbon monoxide; FEF25‐75, forced expiratory flow between 25% and 75% of FVC; FEV1, forced expiratory volume in 1 s; FVC, forced vital capacity; KCO, carbon monoxide transfer coefficient; mMRC, Modified Medical Research Council; n, number.

When cases with and without the BIP pattern were compared, the group with the BIP pattern had a significantly higher smoking burden (32.5 vs. 20 pack‐years; *p* = 0.021). Regarding diffusion capacity parameters, DLCO (57 ± 18 vs. 78 ± 20; *p* = 0.007) and KCO (71 ± 17 vs. 92 ± 16; *p* = 0.002) values were significantly lower in the BIP pattern group. The availability of diffusion capacity measurements was limited, with DLCO and KCO data available for only 33 patients. No statistically significant differences were observed between groups in terms of age, BMI, duration of welding work, or spirometric volume and ratio parameters (FVC, FEV_1_, FEV_1_/FVC, and FEF_25_–_75_) (Table 3).

**TABLE 3:** Comparison of clinical, sociodemographic data, and pulmonary function tests according to the presence of a BIP pattern.

Variables	*n* (BIP positive/negative)	BIP positive (Mean ± SD [%95 CI]/median [IQR])	BIP negative (mean ± SD [%95 CI]/median [IQR])	*p*	Hedges’g
Age (years)	16/57	51.2 ± 7.7 (47.1–55.4)	46.3 ± 9.3 (43.9–48.9)	0.061	0.53
BMI (kg/m^2^)	16/54	27.3 ± 3.7 (25.4–29.4)	27.9 ± 4.0 (26.8–29.0)	0.640	−0.13
Smoking exposure (pack‐years)	14/44	32.5 (23)	20 (20)	0.021	
Duration of welding exposure (years)	16/57	29.2 ± 9.1 (24.3–34.1)	24.0 ± 10.9 (21.1–26.9)	0.086	0.49
FVC (%)	15/54	93 (27)	96 (20)	0.233	
FEV1 (%)	15/54	81 (23)	90.5 (28)	0.111	
FEV1/FVC (%)	15/54	76.1 (9.7)	78.0 (9.9)	0.371	
DLCO (%)	11/22	57 ± 18 (45–69)	78 ± 20 (69–87)	0.007	−1.04
KCO (%)	11/22	71 ± 17 (59–83)	92 ± 16 (85–100)	0.002	−1.23
FEF25‐75 (%)	15/54	61 ± 27 (46–76)	75 ± 29 (67–83)	0.097	−0.48

*Note:* Data are presented as mean ± standard deviation (SD) with 95% confidence intervals (CIs) for normally distributed variables and as median (IQR) for nonnormally distributed variables. For comparisons between groups, an independent samples *t*‐test was used for normally distributed variables, and the Mann–Whitney *U* test for nonnormally distributed variables. Effect size was reported as Hedges’ g for parametric analyses, whereas no effect size was reported for nonparametric analyses. DLCO, diffusing capacity of the lung for carbon monoxide; FEF25‐75, forced expiratory flow between 25% and 75% of FVC; KCO, carbon monoxide transfer coefficient: n, number.

Abbreviations: BMI, body mass index; DLCO, diffusing capacity of the lung for carbon monoxide; FEF25‐75, forced expiratory flow between 25% and 75% of FVC; FEV1, forced expiratory volume in 1 s; FVC, forced vital capacity; KCO, carbon monoxide transfer coefficient; n, number.

The BIP pattern was observed in 13.3% of never smokers and in 16.2% of current smokers; however, the difference in BIP frequency between the two groups was not statistically significant (*p* = 0.102). The proportion of patients with mMRC ≥ 1 was significantly higher in cases with the BIP pattern (*p* = 0.032). Cough was more frequent in the BIP group (31.2%), although the difference was not statistically significant (*p* = 0.089). The presence of concomitant asthma or COPD appeared higher in the BIP group (7/23; 30.4%), but this difference was also not statistically significant (*p* = 0.233). No significant association was found between duration of welding work and the presence of the BIP pattern (*p* = 0.649). Although the prevalence of the BIP pattern was higher among individuals with ≥ 21 years of welding experience (23.8%) compared with those with ≤ 20 years (19.4%), the difference was not statistically significant. Additionally, no significant differences were observed between groups regarding welding type, working in enclosed environments, use of protective masks, or the presence of local exhaust ventilation (LEV) (Table 4).

**TABLE 4:** Distribution of clinical and sociodemographic characteristics associated with the BIP pattern and chi‐square test results.

Variables	Category	BIP positive (*n*[%])	BIP negative (*n* [%])	*p* value
Smoking status	Never smoker	2 (13.3)	13 (86.7)	0.102
	Former smoker	8 (38)	13 (62)	
	Current smoker	6 (16.2)	31 (83.8)	

mMRC score	mMRC score 0	5 (12.5)	35 (87.5)	0.032
	mMRC score ≥ 1	11 (33.3)	22 (66.7)	

Cough	No	6 (14.6)	35 (85.4)	0.089
	Yes	10 (31.2)	22 (68.8)	

Concurrent asthma/COPD	No	9 (18)	41 (82)	0.233
	Yes	7 (30.4)	16 (69.6)	

Duration of welding exposure	≤ 20 years	6 (19.4)	25 (80.6)	0.649
	≥ 21 years	10 (23.8)	32 (76.2)	

Type of welding	Gas metal arc welding (GMAW)	10 (21.2)	37 (78.8)	0.793
	Electrode welding	5 (26.3)	14 (73.7)	
	Other (oxyacetylene, argon welding)	1 (14.3)	6 (85.7)	

Indoor work environment	Yes	14 (20)	56 (80)	0.119
	No	2 (66.7)	1 (33.3)	

Respiratory protective mask	Never used	13 (26.5)	36 (72.5)	0.252
	Irregular use	3 (17.6)	14 (82.4)	
	Regular use	0 (0)	7 (100)	

Local exhaust ventilation (LEV)	Yes	5 (19.2)	21 (80.8)	0.680
	No	11 (23.4)	36 (76.6)	

*Note:* n: number.

Abbreviations: BIP: bronchiolocentric interstitial pneumonia; mMRC: Modified Medical Research Council.

## 4. Discussion

In this study, it was demonstrated that the BIP pattern was detected at a notable frequency among workers occupationally exposed to welding fumes, and that this pattern was associated with certain clinical and functional characteristics. The predominance of nonfibrotic features may suggest that bronchiolocentric abnormalities can be detected at an earlier stage of disease; however, the cross‐sectional design of our study does not allow conclusions regarding temporal progression or reversibility. In the literature, studies systematically evaluating the frequency of the BIP pattern in the context of welding fume exposure are extremely limited; therefore, the descriptive nature of our study becomes particularly important in the interpretation of these findings.

In the existing literature, the BIP pattern has predominantly been discussed in the context of HP. However, accumulating evidence in recent years suggests that bronchiolocentric and airway‐centered radiological patterns are not etiologically limited to HP alone. Indeed, in a recent subgroup analysis evaluating patients with a radiologically “typical HP” pattern, HP was confirmed after multidisciplinary discussion in only approximately one‐third of the 164 patients. An alternative non‐HP diagnosis was identified in 36% of the cases, while no definite etiology could be identified in 34% of the patients [1, 8]. The identified diagnoses included connective tissue disease–associated ILD (notably rheumatoid arthritis), aspiration, and various inhalational or drug exposures. These results show that airway‐centered radiologic patterns are not specific to HP, and interpreting them as HP without broader clinical context may cause misclassification [1, 8, 9]. The findings of our study also suggest that the BIP pattern is not limited solely to immune‐mediated diseases but may also occur in the setting of exposure to inhaled inorganic particles and metal fumes. Indeed, cases of bronchiolocentric fibrotic interstitial involvement associated with the inhalation of metal particles have been reported in the literature. In one case involving exposure to hard metals, pathological examination revealed bronchiolocentric fibrosis, and both clinical and radiological improvement were observed following cessation of the exposure [10]. Furthermore, exposure to welding fumes has been demonstrated in several human and animal studies to cause bronchiolar and peribronchiolar injury and inflammation [6, 11]. The propensity of these particles to deposit in the small airways provides a biologically plausible basis for the development of the BIP pattern through bronchiolocentric inflammation and injury. In this context, the frequent detection of bronchiolar wall thickening and centrilobular ground‐glass nodules on HRCT in our study further supports this proposed mechanism.

These findings can also be considered in the context of the traditional description of pulmonary siderosis. Pulmonary siderosis is generally regarded as a benign pneumoconiosis related to inhalation of iron‐containing particles, with diffuse centrilobular ground‐glass nodules being among its characteristic radiological findings [4, 12]. In our cohort, 7 of the 16 patients fulfilling the operational radiological BIP criteria underwent BAL, and all 7 demonstrated abundant hemosiderin‐laden macrophages (siderophages), while BAL lymphocytes accounted for less than 10% of cells. Together with the characteristic HRCT findings and welding exposure, these findings supported a diagnosis of pulmonary siderosis in these 7 patients. Thus, the radiological BIP pattern observed in our cohort may overlap, at least in some cases, with the traditional radiological and pathological features described in pulmonary siderosis.

Among the patients with available diffusion capacity measurements, DLCO and KCO were significantly lower in cases with a BIP pattern, supporting the potential clinical relevance of the radiological findings. Similarly, previous studies have reported that a reduction in DLCO represents an early and sensitive functional marker in interstitial pneumonias characterized by bronchiolocentric involvement [13–15]. In contrast, although no statistically significant differences were observed in spirometric volume and ratio parameters (FEV_1_, FVC, FEF_25_–_75_), a trend toward lower values was noted. This finding suggests that small airway and peribronchiolar interstitial involvement may not be reflected in spirometry during the early stages, a result that is consistent with the existing literature [13, 15]. Taken together, these findings suggest that the BIP pattern may represent a process that can be detected at an early or even subclinical stage. Nevertheless, the limited sample size should be taken into consideration when interpreting these findings.

A noteworthy point in our study is the relationship between the BIP pattern and smoking exposure. Cigarette smoking can lead to centrilobular ground‐glass nodules, particularly in the context of respiratory bronchiolitis [16], and should therefore be considered a potential confounding factor. In our study, however, smoking status itself (never‐smoker vs. current smoker) was not significantly associated with the presence of the BIP pattern. On the other hand, cumulative smoking exposure was significantly higher among BIP‐positive cases than among BIP‐negative cases (32.5 vs. 20 pack‐years, *p* = 0.021), indicating that a potential contributory or confounding effect of smoking cannot be excluded. Importantly, the presence of centrilobular ground‐glass nodules alone is not specific for smoking‐related respiratory bronchiolitis, and the combination of bronchiolocentric findings used in our operational classification may also occur in other inhalational small‐airway or interstitial processes. Therefore, the observed BIP findings should be interpreted cautiously, taking smoking exposure into account. Given the limited sample size and the small number of BIP‐positive cases, reliable multivariable adjustment for smoking and other potential confounders was not feasible. Accordingly, our findings should not be interpreted as establishing an independent causal association between welding fume exposure and the BIP pattern, but rather as hypothesis‐generating observations that require confirmation in larger studies with appropriate multivariable analyses.

The BIP pattern is not defined within established diagnostic categories such as UIP or NSIP in current guidelines, and its diagnostic boundaries remain uncertain. This ambiguity necessitates that the pattern be interpreted in conjunction with the clinical context, exposure history, and multidisciplinary assessment [1]. Our study adds valuable data on BIP in occupational exposures. Recognizing bronchiolocentric changes on HRCT may raise suspicion for small airway injury from welding fumes, and careful evaluation of occupational exposure history and workplace conditions can help identify potential inhalational causes and guide preventive strategies.

### 4.1. Limitations

This study has several limitations, including its retrospective design, single‐center setting, and lack of histopathological confirmation. Although the study involved specialists from occupational medicine, pulmonology, and radiology, a formal multidisciplinary ILD discussion or multidisciplinary team assessment was not systematically performed for all cases. Importantly, the HRCT‐defined BIP pattern should not be considered equivalent to histopathological BIP. In addition, because there are no universally accepted radiological criteria for distinguishing fibrotic BIP from other fibrotic interstitial pneumonia patterns such as NSIP, some degree of radiological overlap cannot be completely excluded. Furthermore, emphysema was not systematically recorded on HRCT and may therefore have represented an unmeasured confounding factor, particularly in relation to smoking exposure, respiratory symptoms, and pulmonary function parameters. The absence of expiratory CT slices in all cases may have reduced sensitivity for detecting air trapping. Statistical comparisons with the fibrotic BIP group (*n* = 3) were not feasible due to its small size. Nevertheless, the study’s strengths include systematic radiological evaluation of a relatively homogeneous occupational exposure cohort and the integration of occupational medicine, pulmonology, and radiology perspectives.

## 5. Conclusion

This study shows that the BIP pattern is observed at a notable frequency on thoracic CT in welders. To our knowledge, it is the first systematic evaluation of BIP frequency in this group. While the BIP pattern may be associated with welding fume exposure, it should not be regarded as a standalone radiological diagnostic criterion or as equivalent to histopathological BIP. Our findings suggest that a bronchiolocentric interstitial pattern is not exclusive to HP and may occur in association with different inhalational exposures. Therefore, such radiological findings should be interpreted in conjunction with the clinical context, smoking history, detailed occupational exposure history, and, when available, pathological findings and multidisciplinary assessment. These findings are hypothesis‐generating and may provide a basis for future studies investigating the clinical significance and pathological correlates of bronchiolocentric patterns in occupational inhalational exposure.

## Author Contributions

Yusuf Samir Hasanlı: conceptualization, methodology, data curation, investigation, data analysis, writing–original draft, and writing–review and editing, supervision. İpek Candemir: conceptualization, methodology, investigation, writing–review and editing, and supervision. Yeliz Aktürk: methodology, data curation, writing–review and editing, and supervision.

## Funding

This research received no external funding.

## Disclosure

This study was presented as an oral communication at the 29th Annual Congress of the Turkish Thoracic Society (TORAKS 2026), an international congress held on April 8‐12, 2026, in Antalya, Türkiye.

## Ethics Statement

The study was approved by the Ethics Committee of Ankara Etlik City Hospital (approval no: AEŞH‐BADEK1‐2026‐071, Date: January 21, 2026) and conducted in accordance with the Declaration of Helsinki.

## Consent

Due to the retrospective nature of the study, the requirement for informed consent was waived.

## Conflicts of Interest

The authors declare no conflicts of interest.

## Data Availability

The data supporting the findings of this study are available from the corresponding author upon reasonable request.
